# Relationship between ΔSOFA and mortality in patients admitted to the intensive care unit

**DOI:** 10.1186/2197-425X-3-S1-A339

**Published:** 2015-10-01

**Authors:** PC Arvizu-Tachiquín, JA Baltazar-Torres, AA Cano-Oviedo, A Esquivel-Chávez, S Zamora-Varela, NA Canedo-Castillo

**Affiliations:** Intensive Care Unit, Hospital de Especialidades 'Dr. Antonio Fraga Mouret', Mexico City, Mexico

## Introduction

Prognostic scores are important for clinical and administrative management of intensive care units (ICU). SOFA score was designed to describe the sequence of complications during the patient´s stay in the ICU, not to predict outcomes. However, it has been shown that high scores on the SOFA score are associated with high mortality. ΔSOFA is a measure derived from the SOFA score defined as the difference between the SOFA score at admission and the achieved in a determined point of time.

## Objectives

To evaluate the relationship between ΔSOFA and mortality in patients admitted to the ICU.

## Methods

Adult patients of both sexes with stay in the ICU >48 hours were analyzed. Demographic and clinical variables were recorded. The SOFA score was computed on admission and every 48 hours during the stay in the ICU. ΔSOFA was calculated and evaluated as a predictor of mortality through its discriminative capacity and calibration. Logistic regression analysis was performed to identify independent risk factors of death. A p value < 0.05 was considered statistically significant. SPSS 20.0 was used for data analysis.

## Results

Eighty patients, mean age 46 years, 53.8% men were included. The mean length of stay in ICU was 5.11 days and the mortality of 16.3%. The mean SOFA score at admission, maximum and ΔSOFA were 6.01, 6.27 and 0.26, respectively. ΔSOFA showed good discriminative capacity and calibration for predicting mortality (area under the ROC curve 0.914, 95%CI 0.881-1.0, p < 0.05; Hosmer-Lemeshow Chi^2^ 0.371, p = 0.96) and is a risk factor for death.

## Conclusions

ΔSOFA is useful in predicting mortality in patients admitted to the ICU.
